# Item

**Published:** 1907-07

**Authors:** 


### ITEMS.

DR. S. A. KNOPF'S International prize essay, entitled "Tuberculosis as a Disease of the Masses and How to Combat it," a notice of which we published in the June issue of this JOURNAL, page 713, can be obtained from the publisher, F. P. Flori, 514 east eighty-second street, New York, also at Charities, 105 East twenty-second street, New York. The price, paper bound, is twenty-five cents; cloth, fifty cents. We make this announcement at the request of Dr. Knopf.

THE State Civil Service Commission will hold examinations July 13, 1907, to fill among others the following positions: Assistant in clinical laboratory, Manhattan State Hospital, $900 and maintenance; medical inspector of factories, $2,400; superintendent of State School for the Blind, $2,500 and maintenance; trained nurse, state institutions (men and women), $420 to $600 and maintenance; woman physician, state hospitals and institutions, $1,000 and maintenance.

The last day for filing applications for these positions is July 6. Full information and application forms may be obtained by addressing the chief examiner of the commission in Albany, Charles S. Fowler.

